# Sublethal Microcystin Exposure and Biochemical Outcomes among Hemodialysis Patients

**DOI:** 10.1371/journal.pone.0069518

**Published:** 2013-07-24

**Authors:** Elizabeth D. Hilborn, Raquel M. Soares, Jerome C. Servaites, Alvima G. Delgado, Valéria F. Magalhães, Wayne W. Carmichael, Sandra M. F. O. Azevedo

**Affiliations:** 1 United States Environmental Protection Agency, Office of Research and Development, National Health and Environmental Effects Research Laboratory, Research Triangle Park, North Carolina, United States of America; 2 Laboratory of Ecophysiology and Toxicology of Cyanobacteria, Carlos Chagas Filho Biophysics Institute, Federal University of Rio de Janeiro, Rio de Janeiro, Brazil; 3 Department of Biological Sciences, Wright State University, Dayton, Ohio, United States of America; Rutgers University, United States of America

## Abstract

Cyanobacteria are commonly-occurring contaminants of surface waters worldwide. Microcystins, potent hepatotoxins, are among the best characterized cyanotoxins. During November, 2001, a group of 44 hemodialysis patients were exposed to microcystins via contaminated dialysate. Serum microcystin concentrations were quantified with enzyme-linked immunosorbent assay which measures free serum microcystin LR equivalents (ME). We describe serum ME concentrations and biochemical outcomes among a subset of patients during 8 weeks following exposure. Thirteen patients were included; 6 were males, patients’ median age was 45 years (range 16–80), one was seropositive for hepatitis B surface antigen. The median serum ME concentration was 0.33 ng/mL (range: <0.16–0.96). One hundred thirty nine blood samples were collected following exposure. Patients’ biochemical outcomes varied, but overall indicated a mixed liver injury. Linear regression evaluated each patient’s weekly mean biochemical outcome with their maximum serum ME concentration; a measure of the extrinsic pathway of clotting function, prothrombin time, was negatively and significantly associated with serum ME concentrations. This group of exposed patients’ biochemical outcomes display evidence of a mixed liver injury temporally associated with microcystin exposure. Interpretation of biochemical outcomes are complicated by the study population’s underlying chronic disease status. It is clear that dialysis patients are a distinct ‘at risk’ group for cyanotoxin exposures due to direct intravenous exposure to dialysate prepared from surface drinking water supplies. Careful monitoring and treatment of water supplies used to prepare dialysate is required to prevent future cyanotoxin exposure events.

## Introduction

Warm, stable, eutrophic conditions favour growth of cyanobacteria in freshwater systems and cyanobacteria are commonly-occurring contaminants of surface waters worldwide. The effects of climate change and global human population growth with resultant surface water quality degradation directly and indirectly support the increased occurrence of cyanobacteria and their toxins. Microcystins are one of the best characterized groups of cyanotoxins. They are low molecular weight hepatotoxic polypeptides of varying potency; the primary mode of action is as protein phosphatase inhibitors. Microcystins are well documented as a cause of wildlife and livestock mortality events, but documented human exposures are uncommonly reported.

End stage renal failure (ESRF) leaves affected patients with little or no kidney function. Hemodialysis is one method to partially replace kidney function and has proved to be an effective life prolonging treatment for ESRF patients worldwide. During hemodialysis treatment, a patient’s blood is continuously extracted and flows through an extracorporeal dialyzer within which blood flows along a semi-permeable membrane; on the other side of the membrane is a prepared dialysate solution which is maintained at a lower hydrostatic and osmotic pressure. During the hours of hemodialysis treatment, excess intravascular fluid and endogenous toxic waste products such as urea and phosphate are removed from the patient’s circulation. Cleansed blood is continuously returned to the patient’s circulatory system. The frequency and length of treatment and the specific formulation of dialysate solution is part of a customized treatment regimen developed for each patient’s needs [1]. A large volume of dialysate solution is required during each treatment session and is prepared from treated and sanitized fresh water. The semi-permeable membrane, the osmotic and the pressure gradients favor the flow of small-diameter molecules and water out of the patient’s circulation and into the dialysate, although dialysate itself may also enter the patient circulation [2]. Unfortunately, when dialysate solution becomes contaminated, small molecular weight contaminants may pass from the dialysate into a patient’s circulation.

Treatment failures may occur at multiple points in the process when dialysate is prepared from a water source such as municipal drinking water. Microcystins are sporadically reported as contaminants of dialysis treatment [3], [4]. In Brazil, since 2001, dialysate is required to be prepared from drinking water using reverse osmosis (RO) as a final treatment step to protect hemodialysis patients from toxin exposures. During late November 2001, a cyanobacteria bloom occurred in the Funil Reservoir and microcystin concentrations of 0.4 ug/L were measured in treated drinking water distributed in Rio de Janeiro, Brazil.

A survey of dialysis clinics receiving the contaminated drinking water revealed microcystins in dialysate after the final RO process [4]. On December 3, at the Clementino Fraga Filho Hospital (HUCFF) dialysis center at the Federal University of Rio de Janeiro, microcystin concentrations of 0.32 ug/L were measured in the activated carbon filter used as an intermediate step to treat the drinking water supply to prepare dialysate. A group of 44 HUCFF dialysis patients were potentially exposed to microcystin-contaminated dialysate during this cyanobacteria bloom event [4]. A subset of these patients was recruited for longitudinal study to characterize the health effects associated with low dose microcystin exposure; this report describes clinicopathological biochemical outcomes among this group of patients.

## Methods

A subset of hemodialysis patients were selected for inclusion into the longitudinal follow-up portion of the study. Patients were included if on a first blood collection serum microcystin concentrations were above the limit of detection (LOD) of the enzyme-linked immunosorbent assay (ELISA) of ≥0.16 ng/mL of free microcystin-LR equivalents (ME). Subsequent serum samples were collected, frozen to −20°C and analyzed as previously described [5]–[7].

Patients received hemodialysis either on a Monday, Wednesday, Friday (M, W, F) schedule or a Tuesday, Thursday, Saturday (Tu, Th, Sa) schedule. Characteristics of each patient including age, gender, and weight were recorded. Clinical characteristics were recorded for each patient: months receiving dialysis and hepatitis B surface antigen serostatus. Patient’s blood samples were generally drawn during the start of a dialysis treatment. Multiple blood collections per patient and sometimes per week occurred during the study period of December 5, 2001 through January 29, 2002.

All clinicopathological biochemical assays were performed in the clinical laboratory of HUCFF. These included assays that evaluated: hepatic cellular injury: gamma glutamyl transferase (GGT), alanine aminotransferase (ALT), aspartate aminotransferase (AST); cholestasis: GGT, alkaline phosphatase (ALP); hepatic function: total and direct bilirubin (BT, BD); hepatic function and a component of lipid metabolism: triglycerides (TGL); hepatic function and an evaluation of secondary hemostasis: prothrombin time (PT) with its international normalized ratio (INR) [8]. Although a total of 9 biochemical outcomes were included in this analysis, not all biochemical assays were able to be conducted on each blood sample collected from each patient.

### Ethics Statement

Human subjects were recruited and participated in this study during an investigation of a public health event – the exposure of patients to cyanobacterial toxins. Collected data were analyzed in compliance with human subjects policies and procedures of the HUCFF of the Federal University of Rio de Janeiro, and approved by the Institutional Review Board of the University of North Carolina School of Medicine. Dialysis patients gave oral consent to participate in the study. The use of oral consent was approved by the Director of HUCFF, the Dialysis Service of HUCFF and the Health Secretariat of Rio de Janeiro State. Oral consent was documented by the Dialysis Service of the HUCFF.

### Statistical Analysis

Descriptive and statistical analyses were performed using SAS 9.2 analytic software (SAS Institute, Inc., Cary, North Carolina). We evaluated serum ME concentrations by category of subject characteristics, using natural categories where available (male/female; day of dialysis; death during study, etc.) or by categorizing above and below the median value for continuous variables. Geometric mean differences in the maximum serum ME among categories were tested using t-tests on the log-transformed data. The p-values for the t-tests and associated confidence intervals used the pooled variance and were calculated using the SAS GLM regression procedure. Weekly mean, median, and range of values of each biochemical outcome were evaluated among patients and summarized by week; biochemical outcome descriptive statistics were plotted against the HUCFF reference range for each outcome. Serum ME concentrations below the limit of detection (<0.16 ng/mL), were valued at 0.10 to calculate the mean of these values.

**Figure 1:**
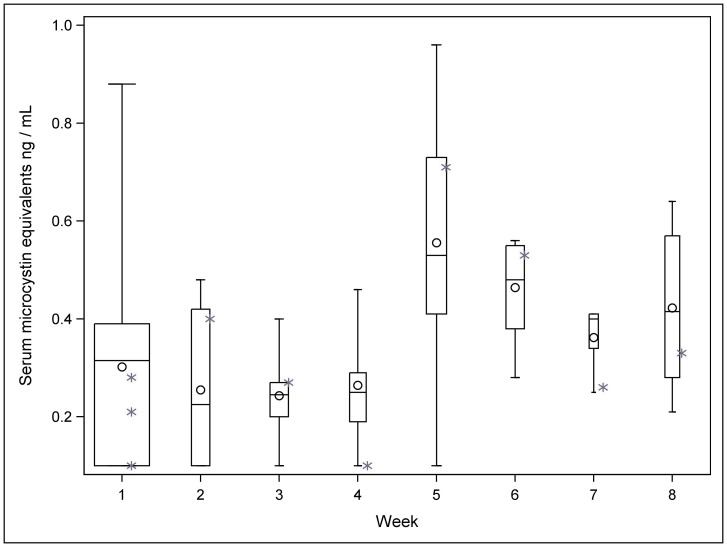
Maximum serum microcystin LR equivalent concentrations ng/mL by week of study, mean (circle), median (line), first - third quartiles (box), minimum, maximum values (whiskers). Values for the one hepatitis B seropositive patient are shown as asterisks and not included in the boxplots. Width of box is relative indicator of number of samples included in analysis each week.

For the purposes of regression analysis, the continuous variables including all biochemical outcomes, serum ME concentrations, body weight in kilograms and total months receiving dialysis were log transformed to better approximate a normal distribution. Serum concentrations of BD included zero values and 0.05 was added to the reported serum concentrations of BD before taking the log. We included the maximum serum ME concentration detected in each patient as the final toxin exposure variable in regression models due to the variability among repeated serum ME concentrations over time among study participants (Figure 1).

Regression was used to evaluate the relationships between weekly mean biochemical outcomes, maximum serum ME and patient characteristics. Two models were specified for each biochemical outcome: 1) simple univariate regressions were performed with each biochemical outcome and the maximum serum ME concentration; and 2) the SAS GLMSELECT stepwise selection procedure was used to specify separate multivariable linear models for each biochemical outcome with all six patient clinical variables and patient characteristic variables evaluated for inclusion in each model as covariates. To account for missing patient characteristic and clinical variable values in the multivariable model, three models were fit for each biochemical outcome: Model 1) covariates with no missing values included gender, day of dialysis, and an indicator for the patient that died during the study; Model 2) covariates from Model 1, plus covariates with a missing value for one patient: age, months of dialysis, and body weight; Model 3) all covariates, including hepatitis B serostatus with missing values in two patients. The final model was selected as follows: Model 3 was selected if hepatitis B serostatus was significant at the 10% level; Model 2 was selected if age, months of dialysis, or body weight was significant at the 10% level; otherwise Model 1 was selected if any of the other covariates were significant at the 10% level. As the main exposure of interest, maximum serum ME was included in every final model regardless of a statistically significant association with the biochemical outcome.

## Results

Of 44 dialysis patients, 13 met the criteria for serum ME concentrations above the limit of detection at the first blood collection and were recruited for follow up. These patients were systematically followed over a total of 56 days (8 weeks). Patients included 6 males and 7 females with a median age of 44.5 years (range 16–80). Of the 11 patients with Hepatitis B serostatus results recorded, one was seropositive to the HBsAg antigen. Seven of the patients received hemodialysis on a M, W, F schedule, and six on a Tu, Th, Sa schedule. The median serum ME concentration among study patients was 0.33 ng/mL, (range: <0.16–0.96 ng/mL) (Figure 1). The maximum serum ME concentration detected among patients was a median of 0.61 ng/mL, (range: 0.31–0.96 ng/mL) (Table 1). A total of 139 blood samples were collected from these 13 patients during the study period.

**Table 1:** Demographic and subject characteristics of 13 hemodialysis patients.

Subject Characteristics	Number (%)
**Gender**, number male, (% male)	**6 (46%)**
**Age in years**, median (range)*	**45 (16–80)**
**Weight in kg**, median (range)*	**59 (45–85)**
**Months of dialysis**, median (range)*	**5 (1–8)**
**Scheduled day of dialysis**, day (%) **M, W, F**	**7 (54%)**
**Hepatitis B**, number seropositive, (%)**	**1 (9%)**
**Death during study**, number (%)	**1 (8%)**
**Maximum serum microcystin concentration** median, (range) **ng/mL**	**0.61 (0.31–0.96)**

* Missing information on one patient.

** Missing information on two patients.

We did not detect any patient characteristic that was associated with significantly higher serum ME concentrations (Table 2). One patient died during week three of the study period; the cause of death is unknown to us. We did not detect higher maximum serum ME concentrations in the patient who died during the study period (0.61 ng/mL), nor in the one patient with serological evidence of Hepatitis B: (0.71 ng/mL); their observed maximum serum ME concentrations were within the group range.

**Table 2:** T-test, geometric mean of maximum serum microcystin LR equivalent concentrations by subject characteristic.

Subject Characteristics	Maximum Serum Microcystin ng/ml,Geometric mean, (95% C.I.)	p-value
**Gender,** (male/female)	**0.62** (0.47, 0.82)/**0.57** (0.44, 0.74)	0.67
**Age in years,** (</> median, 45 years)	**0.66** (0.50, 0.86)/**0.57** (0.43, 0.74)	0.41
**Weight in kg,** (</> median, 59 kg)	**0.68** (0.52, 0.88)/**0.55** (0.42, 0.71)	0.23
**Months of dialysis,** (</> median, 5 months)	**0.57** (0.42, 0.77)/**0.64** (0.50, 0.83)	0.55
**Scheduled day of dialysis,** (M, W, F/Tu, Th, Sa)	**0.53** (0.42, 0.67)/**0.68** (0.52, 0.87)	0.15

### Biochemical Outcomes

**Figure 2:**
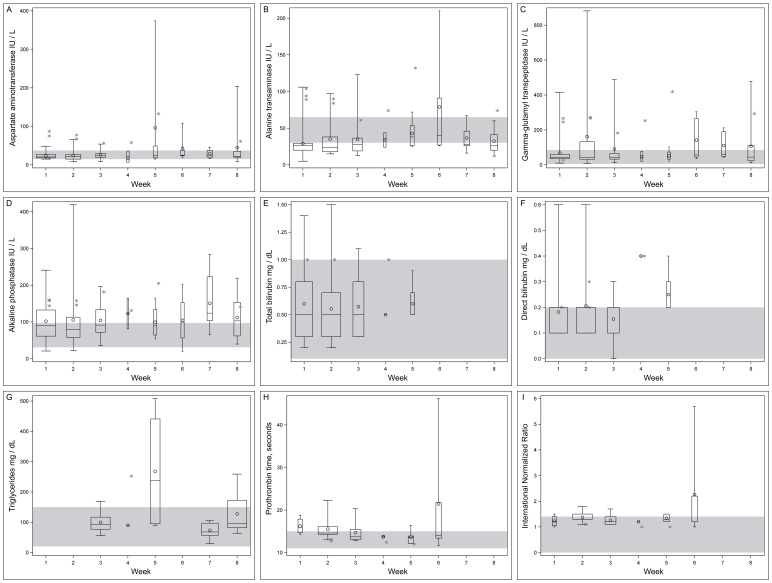
Clinicopathological biochemical outcomes among patients by week of study, a) aspartate aminotransferase, b) alanine aminotransferase, c) gamma-glutamyl transpeptidase, d) alkaline phosphatase, e) total bilirubin, f) direct bilirubin, g) triglycerides, h) prothrombin time, i) international normalized ratio. Mean (circle), median (line), first - third quartiles (box), minimum, maximum values (whiskers). Values for the one hepatitis B seropositive patient are shown as asterisks and not included in the boxplots. Width of box is relative indicator of number of samples included in analysis each week. Shaded area is reference range for laboratory.

The greatest numbers of blood samples were collected during weeks 1–3 (Table 3). Subsequent weeks yielded fewer samples; this precluded a repeated measures analysis of biochemical outcomes within each patient’s collection of serum samples over the study period. However, we display each biochemical outcome distribution among patient samples by week over the study period in the context of reference ranges for each biochemical outcome (Figure 2).

**Table 3:** Weekly serum microcystin LR equivalent and biochemical outcomes, descriptive statistics.

Biochemical Outcome	n*	Minimum value	Maximum Value	Mean	Median
Aspartate aminotransferase (AST), Reference range: 15–37 IU/L	93	7.0	374.0	35.6	24.0
Week 1	27	15.0	87.0	27.2	21.0
Week 2	21	8.0	77.0	28.5	22.0
Week 3	13	8.0	56.0	28.3	24.0
Week 4	4	7.0	58.0	28.5	24.5
Week 5	6	15.0	374.0	102.2	36.5
Week 6	5	20.0	108.0	43.0	25.0
Week 7	7	17.0	46.0	29.4	25.0
Week 8	10	9.0	204.0	46.4	22.5
Alanine transaminase (ALT), Reference range: 30–65 IU/L	93	5.0	210.0	41.2	28.0
Week 1	27	5.0	106.0	36.4	27.0
Week 2	21	15.0	97.0	39.9	26.0
Week 3	13	13.0	123.0	36.8	29.0
Week 4	4	24.0	74.0	44.0	39.0
Week 5	6	25.0	132.0	57.8	46.0
Week 6	5	26.0	210.0	78.8	40.0
Week 7	7	16.0	67.0	36.4	28.0
Week 8	10	12.0	74.0	36.4	30.0
Gamma-glutamyl transpeptidase (GGT), Reference range: 5–85 IU/L	90	7.0	882.0	116.9	48.0
Week 1	28	10.0	416.0	79.4	41.5
Week 2	21	7.0	882.0	171.8	55.0
Week 3	13	13.0	488.0	97.8	46.0
Week 4	4	21.0	253.0	98.0	59.0
Week 5	5	21.0	419.0	126.2	45.0
Week 6	5	35.0	304.0	141.2	57.0
Week 7	5	43.0	212.0	110.4	60.0
Week 8	9	12.0	479.0	126.8	49.0
Alkaline phosphatase (ALP), Reference range: 31–97 IU/L†	87	20.0	419.0	114.1	104.0
Week 1	27	21.0	241.0	107.7	91.0
Week 2	19	22.0	419.0	110.4	81.0
Week 3	13	36.0	196.0	110.3	99.0
Week 4	3	81.0	165.0	125.7	131.0
Week 5	5	54.0	205.0	120.6	104.0
Week 6	4	20.0	203.0	104.5	97.5
Week 7	7	65.0	284.0	150.7	124.0
Week 8	9	40.0	218.0	115.1	106.0
Total bilirubin (BT), Reference range: 0.1–1 mg/dL	58	0.20	1.50	0.61	0.50
Week 1	19	0.20	1.40	0.64	0.50
Week 2	21	0.20	1.50	0.60	0.50
Week 3	11	0.30	1.10	0.57	0.50
Week 4	3	0.50	1.00	0.67	0.50
Week 5	4	0.50	0.90	0.60	0.50
Direct bilirubin (BD), Reference range: 0–0.2 mg/dL	57	0.0	0.60	0.20	0.20
Week 1	19	0.10	0.60	0.18	0.20
Week 2	20	0.10	0.60	0.21	0.20
Week 3	11	0.00	0.30	0.15	0.20
Week 4	3	0.40	0.40	0.40	0.40
Week 5	4	0.20	0.40	0.25	0.20
Triglycerides (TG), Reference range: 20–150 mg/dL	29	30.0	508.0	129.2	92.0
Week 3	8	56.0	169.0	99.6	92.0
Week 4	2	90.0	252.0	171.0	171.0
Week 5	4	90.0	508.0	268.0	237.0
Week 7	7	30.0	106.0	73.6	68.0
Week 8	8	64.0	259.0	127.8	95.0
Prothrombin time (PT), Reference range: 11–15 seconds	48	11.7	46.2	15.5	14.2
Week 1	4	14.3	18.8	16.3	16.0
Week 2	20	12.7	22.3	15.3	14.5
Week 3	10	12.9	20.3	14.7	13.7
Week 4	3	12.4	14.1	13.3	13.4
Week 5	6	12.0	16.4	13.4	12.9
Week 6	5	11.7	46.2	21.4	14.0
International Normalized Ratio (INR) Reference <1.4	48	1.0	5.7	1.4	1.3
Week 1	4	1.0	1.5	1.2	1.2
Week 2	20	1.1	1.8	1.3	1.3
Week 3	10	1.1	1.7	1.3	1.2
Week 4	3	1.0	1.2	1.1	1.2
Week 5	6	1.0	1.5	1.3	1.3
Week 6	5	1.0	5.7	2.3	1.2
Microcystin LR equivalents (ME) ng/mL	103	<0.16	0.96	0.35	0.33
Week 1	33	<0.16	0.88	0.29	0.28
Week 2	11	<0.16	0.48	0.27	0.23
Week 3	11	<0.16	0.40	0.25	0.25
Week 4	10	<0.16	0.46	0.25	0.25
Week 5	12	<0.16	0.96	0.57	0.57
Week 6	11	0.28	0.56	0.47	0.52
Week 7	6	0.25	0.41	0.35	0.37
Week 8	9	0.21	0.64	0.41	0.40

† Reference range [36]; all other reference ranges provided by the Clementino Fraga Filho Hospital Laboratory.

* Not every assay was performed on each blood sample.

Patients’ biochemical outcomes were frequently observed outside of the reference ranges (Table 3). Among some samples, markers of hepatic cellular injury during weeks 1–8 exceeded the reference range for: AST, ALT and GGT. Mean values of AST exceeded the reference range during weeks 5, 6 and 8, and a maximum value during week 5 was 10× the upper limit of the normal (reference) range (ULN). Mean values of ALT exceeded the reference range during week 6, and a maximum value during week 6 was greater than 3× the ULN. Mean values of GGT, exceeded the reference range overall and during weeks 2–8, and a maximum value during week 2 was greater than 10× the ULN. In general, the patient with positive HBsAg serology was included among those whose values for hepatic cellular injury were recorded above the reference range. Some patient ALT values were observed below the reference range during all 8 weeks, and at least 1 patient’s AST values were observed below the reference range during week 2, 3, 4 and 8.

Values for a marker of cholestasis, ALP, were also observed above the reference range during all 8 weeks; this included the patient with positive HBsAg serology. Mean values of ALP exceeded the reference range overall and during all 8 weeks of the study; median values exceeded the reference range overall and during weeks 3–8. A maximum value of ALP was greater than 4× the ULN during week 2. Patient ALP values were recorded below the reference range during week 1, 2 and 6.

Markers of hepatic function were also evaluated. A single patient’s BT value exceeded the reference range by less than 2× the ULN during weeks 1, 2 and 3; some patient BD values exceeded the ULN during week 1–3; during week 4 and 5 all patient values were at or exceeded the ULN by less than 2×. Mean and median BD values exceeded the reference range during week 4 and mean values during week 5. TG was measured among patient samples during weeks 3, 4, 5, 7 and 8; values exceeded the reference range during weeks 3, 4, 5 and 8, the maximum value was greater than 3× ULN during week 5. Mean and median TG levels exceeded the reference range during weeks 4 and 5. During weeks 1–3, 5 and 6, one or more patients’ PT and INR values exceeded the reference range. Mean and median PT values exceeded the reference range during week 1 and the mean value exceeded the reference range during weeks 2 and 6. The overall mean INR exceeded the reference range, and the mean INR value exceeded the reference range during week 6, with a maximum value greater than 4× ULN (Table 3, Figure 2).

### Regression Analysis

Maximum serum ME concentrations were inversely and significantly associated with PT and its’ INR. Among adjusted models, the co-variates hepatitis B serostatus, body weight and day of the week dialysis was received were found to be significantly associated with biochemical outcomes during the stepwise selection process and were therefore included in specific adjusted models. We report both unadjusted and adjusted results along with covariates included in the final regression models of biochemical outcome and maximum serum ME concentrations (Table 4).

**Table 4:** Regression model parameter estimates of biochemical outcomes by maximum microcystin LR equivalent concentration.

Biochemical Outcome	Model	Parameter for Maximum microcystin			Covariates in adjusted model
		β	Std. Error	p-value	
Alanine aminotransferase (ALT)	Crude	0.34	0.54	0.55	
	Adjusted	0.50	0.47	0.32	Hepatitis B seropositivity
Aspartate aminotransferase(AST)	Crude	−0.43	0.38	0.28	
	Adjusted	−0.31	0.19	0.15	Hepatitis B seropositivity
Gamma glutamyl transferase(GGT)	Crude	−0.72	0.87	0.43	
	Adjusted	−0.66	0.72	0.39	Hepatitis B seropositivity
Alkaline phosphatase (ALP)	Crude	−0.02	0.52	0.97	
	Adjusted	–	–	–	
Total Bilirubin (BT)	Crude	−0.15	0.49	0.77	
	Adjusted	–	–	–	
Direct Bilirubin (BD)	Crude	−0.06	0.42	0.90	
	Adjusted	–	–	–	
Triglycerides (TG)	Crude	−0.54	0.50	0.31	
	Adjusted	−0.33	0.39	0.42	Hepatitis B seropositivity
Prothrombin time (PT)	Crude	−0.28	0.09	<0.01	
	Adjusted	−0.37	0.08	<0.01	Day of week dialysis received
International normalized ratio(INR)	Crude	−0.36	0.11	<0.01	
	Adjusted	−0.28	0.06	<0.01	Hepatitis B seropositivity and body weight

## Discussion

Patients’ biochemical outcomes were frequently observed outside of the HUCFF reference ranges. Among some but not all samples, markers of hepatic cellular injury during weeks 1–8 exceeded the reference range for AST, ALT and GGT. Elevations of AST and ALT are markers of hepatocellular damage. These observations are expected as liver is the primary target organ for microcystin toxicity as the toxin is actively transported into hepatocytes. The mode of action is protein phosphatase inhibition which results in rapid damage to these metabolically active cells [9]–[12].

Other investigators have reported associations between human microcystin exposure and subsequently elevated liver enzymes associated with hepatic cellular injury such as GGT, ALT, AST, BT and those outcomes associated with less specific tissue damage: lactate dehydrogenase (LDH), and with cholestasis: GGT and ALP [13]–[17]. In this study, BT values exceeded the reference range during weeks 1 and 3; some BD values exceeded the reference range during all weeks it was measured. While BT is a relatively insensitive measure of hepatic function, BD increases in response to hepatic biliary dysfunction. An early and persistent increase in ALP and GGT was noted among some patients during all 8 weeks of evaluation. Increased ALP with GGT, and to a lesser extent, BD is associated with cholestasis and biliary dysfunction [18]. Together, these elevations in biochemical markers are indicative of a mixed liver injury, including components of both cholestatic and hepatocellular injury [19].

Microcystins are understood to be eliminated into the small intestine during biliary excretion [20], [21]. The biochemical response of these patients after microcystin exposure is compatible with mild to moderate intrahepatic cholestasis, a restriction of biliary excretion of bile acids. Damage to hepatocytes surrounding canaliculi may mechanically restrict bile flow out of the liver and effect intrahepatic cholestasis. However, there are potentially other mechanisms associated with bile production that may be disrupted in response to toxins [22]. Evaluation of serum bile acid concentrations after human microcystin exposure events may further inform the understanding of this observed phenomenon.

All of the patients included in this study were selected for early, measurable serum ME concentrations. Microcystin concentrations of 0.4 ug/L were measured in the source water and 0.32 ug/L in the activated carbon filter used as an intermediate treatment step to prepare dialysate. Compared to the 1996 Caruaru incident where patients were exposed to much higher microcystin concentrations, an estimated 19.5 ug/L, patients included in this study were exposed to much lower detected doses [6]. We are not aware of any dialysis patients even among our larger exposed group that died with acute hepatic failure as was observed in the 1996 hemodialysis microcystin exposure incident [3].

The results of our regression analysis indicated that a biochemical outcome associated with clotting time (PT and its’ INR) was significantly and negatively associated with maximum serum ME in both crude and adjusted models in this study population. This is a new finding and we are not aware of others evaluating this outcome after human exposure events to microcystins. Physiologically, liver injury is associated with increases in PT and its INR due to disruption in the synthesis of the factor proteins required for normal function of the clotting and fibinolytic systems [23]. Indeed we observe mean PT and INR values above the normal range during week 6, a week when other biochemical makers of liver injury are present and elevated above the reference range.

Hemodialysis patients have, by definition, chronic disease which complicates interpretation of biochemical responses to microcystin exposure. Interpretation of clotting time must include the knowledge that hemodialysis patients experience a relatively hypercoagulable state compared to individuals without renal failure [24]. Anticoagulation therapy is standard during hemodialysis to maintain vascular access. Hemodialysis patients included in this study received heparin but not warfarin. Heparin therapy, unlike warfarin, does not typically alter prothrombin time nor by extension the INR [25]. Therefore, these observed alterations in clotting time are not believed to be associated with dialysis-associated drug therapy. However, observed clotting time abnormalities associated with liver injury may differ between dialysis patients and the general population.

Our regression analysis evaluated biochemical outcomes associated with hepatocellular injury and cholestasis as well. However, other biochemical markers of hepatocellular damage or cholestasis such as AST, GGT and ALP were not always positively associated in regression analysis, nor did any outcomes achieve statistical significance in our small study population.

### Effects Specific to End Stage Renal Failure and Dialysis

Because of the multiple physiologic differences between patients with ESRF and the general population, it is difficult to directly compare the biochemical outcomes of this subset of microcystin-exposed patients to outcomes that may be associated with a similar microcystin exposure in the general population. For example, ESRF patients may display significantly reduced transaminase values in response to hepatic injury when compared to population-based reference ranges or to healthy controls [26]–[28]. This potential for a reduced transaminase elevation should be considered when assessing hepatic injury in a microcystin exposed individual with ESRF.

Mean and median TG values exceeded the reference range during study weeks 4 and 5. Surviving hemodialysis patients involved in the 1996 microcystin intoxication incident who received evaluation after the event also displayed hypertriglyceridemia [14]. Hemodialysis patients typically have higher serum triglyceride concentrations, associated with the oxidative stress and with the heparin therapy associated with hemodialysis [29], [30]. Microcystins are also known to be a source of oxidative stress [31]. The ultimate consequence of the multiple physiologic stressors associated with hemodialysis on triglyceride concentrations is poorly characterized, and it is unknown how microcystins may further alter these processes. Therefore, this finding of hypertriglyceridemia may not be generalizable to other groups of individuals exposed to microcystins but without concurrent ESRF or dialysis treatment.

Viral hepatitis infection is common among dialysis patients and is associated with increased mean transaminase values compared to healthy adults [32]. Direct bilirubin may also be elevated among patients with hepatitis B [18]. Indeed, the one patient with documented HBsAg seropositivity displayed elevated ALT, AST and BD concentrations during most times they were evaluated.

### Limitations

Two patients’ serostatus was unknown for hepatitis B; none were evaluated for hepatitis C. Therefore, some transaminase values may be impacted by an underlying and unrecorded liver disease that is not associated with microcystin exposure.

ELISA is considered a semiquantitative method to measure serum ME concentrations. It appears to measure the variable ‘free’ portion of total microcystins that are present in human serum [7]. Therefore, serum ME concentrations should not be interpreted as the total amount of circulating microcystins.

During the time period of the study, patients continued to have measureable ME in serum and continued elevations of liver tests for weeks after initial microcystin exposure to contaminated dialysate was suspected. No further contamination in dialysate was detected during a weekly evaluation of water samples used for preparation of dialysate during the entire study period. This persistent elevation in serum microcystin concentrations is consistent with observations made almost 90 days after the 1996 intoxication event where hemodialysis patients’ serum microcystin concentrations remained elevated [4]. However, it is possible that study patients were exposed to other undetected sources of microcystins after their initial exposure.

### Summary

This episode represents an unusual, well documented, low dose human microcystin exposure event. This group of exposed patients appears to display evidence of a mixed liver injury temporally associated with microcystin exposure. INR was significantly and negatively associated with maximum serum ME. However, assessment of biochemical outcomes as a reflection of microcystin-associated liver injury is complicated by the study population’s underlying chronic disease states. Serum ME concentrations persisted throughout the study period; it is unknown if this is consistent with microcystin metabolism or if there was an undetected, ongoing source of microcystin exposure.

This study provided more information about the human biochemical response to sublethal microcystin exposure. However, the evaluation of these biochemical outcomes also raised more questions. We recommend that future studies of human microcystin intoxication events include the assessment of not only liver tests but also: coagulation assays; blood lipid panels; glucose; proteins (total, albumin and globulin); bile acid concentrations and longitudinal evaluation of serum microcystin concentrations and biochemical outcomes over time.

It is clear that dialysis patients are a distinct ‘at risk’ population for cyanotoxin exposures due to direct intravenous exposure to dialysate prepared from surface drinking water supplies. It is well documented, that despite water treatment, drinking water can become contaminated with cyanotoxins [33]–[35]. Therefore, any further breakdown in purification processes during the preparation of dialysate may result in a cyanotoxin exposure episode. Hemodialysis patients’ exposure risks to cyanotoxins are potentially very high, and it is prudent to secure a safe, monitored supply of dialysate with multiple barriers to contamination that are closely monitored to prevent future exposure episodes among this vulnerable group.
